# A Positive Emotional-Based Meditation but Not Mindfulness-Based Meditation Improves Emotion Regulation

**DOI:** 10.3389/fpsyg.2019.00647

**Published:** 2019-03-28

**Authors:** Camila P. R. A. T. Valim, Lucas M. Marques, Paulo S. Boggio

**Affiliations:** Social and Cognitive Neuroscience Laboratory and Developmental Disorders Program, Center for Health and Biological Sciences, Mackenzie Presbyterian University, São Paulo, Brazil

**Keywords:** meditation, emotion regulation, mindfulness, cognitive reappraisal, interbeat-interval

## Abstract

Among the various strategies for modulating the components of the emotional responses, the cognitive reappraisal and distraction are highlighted in current researches. As indicated in recent studies, the capacity for emotional regulation can be improved by mindfulness meditation practicing. This practice usually offers benefits to people’s cognitive functioning and aims to improve a characteristic that is intrinsic to every human being: the ability to turn attention to the present moment. Importantly, positive emotions might also be effective on emotional regulation and several meditation practices make use of it. Thus, we aimed to compare two meditation modalities: one focused on attention only (mindfulness) and another focused-on attention toward positive emotions [Twin Hearts Meditation (THM)]. Ninety healthy subjects without any previous experience in meditation were enrolled in this experiment. Of these participants, 30 were submitted to the mindfulness practice with full attention on the observation of thoughts; 30 to the THM; and 30 to a control group (no meditation practice). After one session of meditation, all the participants completed emotional regulation task judging the valence and arousal of pictures with emotional content. In addition to the behavioral data, the participants’ psychophysiological measures were recorded via electrocardiography (ECG). The results demonstrate a greater efficacy of THM in suppressing the negative valence of the negative pictures and amplifying the valence of the positive ones. No effect of meditation was observed for the ECG. Our findings indicate that contemplative meditation (THM) can positively influence the emotion regulation ability, even when performed by non-meditators and only once. However, in mindfulness meditation this same immediate effect was not found. Our findings reveal that faster effects of meditation practices can be obtained by practices that considers either the attentional processing and the positive emotions.

## Introduction

Current studies characterize emotion as a complex phenomenon arising from responses to external and internal stimulus (mental representations) of the individual (Barrett et al., 2016). This phenomenon appears when a person comes into contact with a relevant situation (Gross and Thompson, 2006; Gross, 2014) and can manifest itself in physiological, behavioral and subjective responses (Gross and John, 2003).

For a better understanding of how the phenomenon of emotion occurs, Gross and Thompson (2006) have organized a scheme that explains emotional processing. This scheme, denominated *modal model*, was elaborated in order to unite the convergent points among several researchers about emotion topic and can be divided into four stages, which are: (1) Situation; (2) Attention; (3) Evaluation; and (4) Response.

Considering this emotion-modulating characteristic, some studies investigate strategies of emotion regulation, in other words, ways of modulating emotional response components (Gross, 2002). This modulation, however, does not aim at modifying the valence of emotion (transforming sadness into joy, for example), but typically changes its dynamics, increasing; decreasing; or maintaining the intensity of the emotional response (Gross and Thompson, 2006). The present study focuses on two of the emotion regulation strategies that fit into the group of strategies antecedent to the response, which are: *distraction* (or attentional deployment) and *cognitive reappraisal* (or cognitive change). Distraction involves the use of selective attention to regulate the effects of an emotional response (McRae et al., 2010). In other words, the modulation of attention is used to change the intensity of the emotional response. This strategy is also part of the so-called “disengagement strategies,” which are those whose attentional focus is drawn from the emotional stimulus to an alternative stimulus (Sheppes et al., 2011). The reappraisal strategy, in turn, aims to change the trajectory of the emotional response, as the stimulus label is reinterpreted (McRae et al., 2010). Thus, before a stimulus, the use of reappraisal can decrease the emotional arousal (Down-regulation) that a given situation would cause if there were no cognitive transformation proposed by this strategy (Gross, 2002), as well as increase (Up-regulation) this excitation.

From a practical point of view, there is a concern to study the effects of emotion regulation on people’s daily lives and, consequently, the importance of this cognitive function on emotional well-being (Brockman et al., 2017). Studies on this topic have already shown their great relevance to the clinical context. According to the review elaborated by Berking and Wupperman (2012), the misuse of strategies of emotion regulation is positively related to neuropsychiatric disorders, such as: borderline personality disorder (Schulze et al., 2011); depression (Ehring et al., 2008); anxiety disorder (Cisler et al., 2010); and substance abuse (Baker et al., 2004). In this sense, it becomes evident the importance of other studies that focus on emotional regulation and it impacts on individuals’ mental health.

In addition to emotion regulation, meditative practices have also been associated with emotional well-being (Fredrickson et al., 2017). Among them, mindfulness (MF) meditation is the most investigated one (Ortner et al., 2007; Fredrickson et al., 2017). This practice, rooted in Buddhist philosophy, can be understood as a systematized strategy of refining a mode of consciousness intrinsic to every human being: the ability to turn attention to the present moment without judgment, reaction or interference (Kabat-Zinn, 2015). Thus, MF is considered an attribute of consciousness that uses two other important components of consciousness itself: awareness and attention. The first can be understood as the component that keeps a broad focus on everything that comes or goes out of consciousness. On the other hand, attention is understood as the process of directing the focus to a specific stimulus or experience (Brown and Ryan, 2003). In this sense, MF results in the process of directing awareness to an experience that occurs in the present, with the aid of the regulation of sustained attention in this focus (Garland et al., 2009). MF meditation is typically described as a practice that brings benefits to the clinical disorders treatment from a various origin, from neuropsychiatric conditions to physical health disorders, such as anxiety and cortisol levels reduction (Hölzel et al., 2016). For this reason, this practice has gained wide prominence in empirical research in the last years, in addition to the clinical practice (Gu et al., 2015).

According to Buddhist philosophy, emotion regulation forms another important aspect of MF, which is the reduction of the negative and increase of positive emotions, what moves toward an “awakening of consciousness and a positive state of being” (Trungpa, 1984; Garland et al., 2009). The two concepts still resemble the very purpose of emotional regulation: to increase the chances of an emotion being favorable and not harmful by modulating it (Gross, 2015). Empirically, the cognitive capacity for emotion regulation seems to really be enhanced from the practice of MF. Opialla et al. (2015), in a picture observation/evaluation task, compared a group who were instructed to perform cognitive reappraisal to another instructed to perform MF. The results of functional magnetic resonance imaging (fMRI) demonstrated that both strategies involved similar neural circuits, namely: prefrontal cortex and amygdala.

Previous studies have already investigated that during the process of emotion regulation, the activation of the prefrontal cortex decreases amygdala activity (Ochsner et al., 2004). Similarly, a neuroimaging study that investigated the effect of the disposition for MF in brain areas predicted that during a task of identifying emotional content in facial expressions, higher levels of this disposition (as measured by the Mindful Attention Awareness Scale; Brown and Ryan, 2003) increased activation of the prefrontal cortex, as well as inhibited amygdala activity (Creswell et al., 2007). In addition to findings from neuroimaging studies, the relation between MF and emotion regulation has also been investigated in the psychophysiological context. In this sense, Pavlov et al. (2015) conducted a study in which an group of 20 experienced meditators was compared to a control group, during a picture observation/evaluation task of positive and negative related pictures. The behavioral data and the recording of the electrocardiographic activity demonstrated positive influence of the practice of MF in emotional control.

It is important to note that MF is not the only type of meditation that has been associated with benefits to people’s lives. In general, meditative practices promote changes in the psychological and personal experience of practitioners (He et al., 2015). The Loving-Kindness Meditation (LKM), for example, is a type of Buddhist meditation that proposes to the practitioner to cultivate unconditional kindness for oneself and for others. As a result, LKM amplifies and cultivates positive valence emotions (Zeng et al., 2015). Similar to this type of meditation, other practices are also grounded in the development of kind and altruistic attitudes, such as compassion meditation and contemplative meditations (e.g., Christian contemplation). Galante et al. (2014), in their revision, denominate these types of practices like belonging to the group of Kindness-Based Meditation (KBM), being LKM the best known and used. Thus, specifically about LKM, its practice typically consists of repeating phrases such as “may you be happy and free from your suffering” or the visualization of a light emanating from the practitioner to others, representing feelings of kindness and love (Galante et al., 2014; Zeng et al., 2015).

In this same purpose, there is another meditative practice little known and not yet explored scientifically, whose characteristics very much resemble LKM. Named Twin-Hearts Meditation (THM), this practice was created by Grandmaster Choa Kok Sui, a Filipino of Chinese origin who has his work known as “Pranic Healing” (alternative medicine of healing with hands). Like the LKM, THM also draws attention to positive emotions, since the participant is encouraged to imagine the Earth planet in front of him, mentalize positive thoughts and direct them toward humanity.

Considering that previous studies have already investigated the influence of MF on emotion regulation, showing this relationship more specifically with the strategy of cognitive reappraisal (Krygier et al., 2013), it is interesting to investigate the effect of this type of meditation in distraction strategy. In addition, it is relevant to compare this meditation of already known effect in the emotion regulation with another meditation whose attentional focus is on the positive emotions, like THM. This research line is congruent with discussions that have been carried out in the current panorama regarding the differences between the strategies of emotion regulation (Sheppes et al., 2011), besides of the most present themes in the current research (Bishop et al., 2004; McRae et al., 2010; Fredrickson et al., 2017) which contribute to the well-being and quality of life of people (Brown and Ryan, 2003; Berking and Wupperman, 2012).

In this sense, this study aimed to investigate the behavioral effects (see the section “Emotion Regulation Task” in “Materials and Methods”) and psychophysiological (see the section “Interbeat Interval Measure” in “Materials and Methods”) of two different meditation practices (MF and THM) in two strategies of emotion regulation, namely: cognitive reappraisal (up- and down-regulation) and distraction.

## Materials and Methods

### Participants

Ninety healthy adults between 18 and 35 years (22.02 ± 3.73; 55 females) participated in the study. The sample calculation was performed using G^∗^Power software^®^, using the study of Kanske et al. (2010) as reference. We excluded individuals with a history of neuropsychiatric disorders; history of dependence on alcohol and other drugs; and who had experience with any type of meditation, including yoga. Then, participants were **randomly allocated** into one of the three groups (30 participants per group): (G1) Twin Hearts Meditation (THM); (G2) Mindfulness Meditation; (G3) Passive Control Group.

A between-subjects design was employed, controlling for possible practice effects in the task of cognitive reappraisal. The participants recruitment occurred through ads on social networks focused on university students. All experimental procedures described herein have been approved by the local ethics committee (SISNEP, Brazil; CAAE: 79465017.7.0000.0084).

### Experimental Procedure

After the arrival of the participant in the lab, it was explained to **him/her** the purpose of the study, tasks and procedures that would be used, as well as all possible risks and benefits of the experiment. Then, in the experimental room the participant gave his consent and answered the Brazilian version of the following instruments: (i) The Emotion Regulation Questionnaire (ERQ), with both subcomponents, the ERQ-CR and ERQ-S [Cognitive Reappraisal and Suppression, respectively (Boian et al., 2009)]; (ii) Beck Anxiety Inventory (BAI; Gorenstein and Andrade, 1996); (ii) Beck Depression Inventory (BDI; Gorenstein and Andrade, 1996); Positive and Negative Affect Scale (PANAS; Siqueira et al., 1999); and the Philadelphia Mindfulness Scale (PMS; da Silveira et al., 2012). All these scales were used to control for possible baseline differences between groups. The specific procedure relative to each group intervention are described in the next topics.

After the meditation intervention, participants completed the PANAS scale once more and were positioned comfortably seated approximately 1 m from the monitor. The sites for electrocardiographical (ECG) electrodes positions were cleaned with ethanol solution, followed by the placement of the electrodes. After that, participants from all groups performed the Emotion Regulation Task. Finally, the electrodes were removed from the participant. At this stage, the participants completed the PANAS scale a third time. All participants received university credits for their participation. The entire procedure took approximately 2 h.

#### G1 – Twin Hearts Meditation

Considering the various types of contemplative meditation, it was chosen the THM. The instruction of this practice was performed by a participant of the Uni Prana group (school of Pranic Healing and Arhatic Yoga of the Grandmaster Choa Kok Sui), who was able to instruct the procedures of the THM practice. This practice, in turn, lasted 27 min and was presented to the participant by an instructional audio. Participant were constantly instructed to desire good things for the Earth and for **all humanity**, invited to imagine the Earth in front of him, to place his hands toward her and then to repeat positive words and phrases to himself. In this meditation, attention is directed toward this exercise of gratitude.

The instructor then briefly explained and standardized the purpose of the meditation and directed the participant to sit with his feet flat on the ground, standing upright and not resting on the back of the chair. After participant positioned himself in this way, the instructor started the audio and then leave the participant alone in the experimental room practicing.

#### G2 – Mindfulness Meditation

The MF practice, similar to the meditation in the G1, was instructed to the participant through a 20-min audio. For this group, the researcher (who was trained to instruct this meditation) explained the purpose of the practice and gave the standardized guidelines of how the participant’s posture should be throughout the meditation (exactly as instructed in the G1). This practice, differently from G1, aimed to direct the participant’s attention to the observation of his own thoughts, perceiving their arrival and letting them go. As a classic MF practice (i.e., focusing on breathing or bodily sensations), the practice of observing thoughts also aims to regulate attention to a specific stimulus (in this case, thoughts) without reacting or being affected by then. Throughout the meditation, the participant was invited to keep his attention in this focus, returning whenever he became distracted.

#### G3 – Control Group

For the control group, the same time used by the other two groups was adopted for an accomplishment of their own meditations, with a difference that participants under control group should remain at rest for the entire period, without performing any kind of activity (even using smartphones for any purpose), until the end of the 20 min. This group was included as a passive control group for the two types of active intervention (groups 1 and 2).

### Emotion Regulation Task

**Figure 1:**
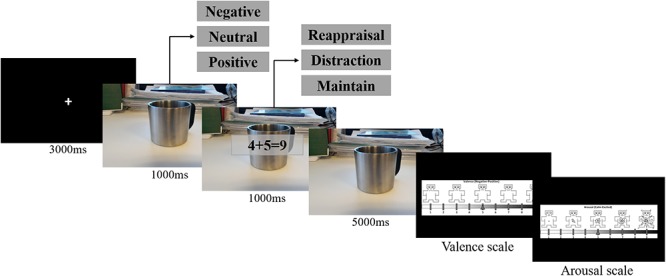
Representation of one single trial for each emotion regulation strategy and each emotional valence condition.

An experimental design similar to that developed by Kanske et al. (2010) was used, as well as the same emotional stimuli chosen in their study. In this sense, 48 pictures were selected from the International Affective Picture System (IAPS; Lang et al., 2005). The selected pictures were characterized by 16 positive, 16 negative, and 16 neutral (pictures information is provided as Supplementary Material). The experimental task (Figure 1) occurred as follows: the participant observed a pseudo-randomized sequence of emotional pictures. Each group of images (negative, positive, or neutral) appeared in three different emotion regulation conditions/strategies, namely: cognitive reappraisal strategy; distraction strategy; and maintain strategy (passive observation).

Under cognitive reappraisal strategy, participants were observed the pictures alone in the screen (1,000 ms) and then, in front of the picture, an instructional appeared (as a watermark) instructing how to reappraise the picture content (1,000 ms). If the word “*Decrease*” appears (1,000 ms), the participant should perform down-regulation in the following period of the picture presentation (only the picture for 5,000 ms without watermark), i.e., decreasing the picture emotional arousal. To do this, before starting the task, the participant was instructed on how to carry out such a strategy. In this sense, it was suggested that the participant should think that the situation represented by the image was a passing situation that would decrease in intensity (same instruction given to all participants). If the word that appeared on the screen was “*Increase*” (1,000 ms), the participant should perform the Up-regulation, increasing the current emotional arousal. In this case, it was suggested to the participant to imagine that the presented situation would be even more intense. The participant should perform Up-regulation in the following period of the picture presentation (5,000 ms). It is important to note that Down-regulation reappraisal was only presented under negative pictures trials as well as Up-regulation reappraisal was only presented under positive pictures trials. The idea was to increase the impact of positive situations while decreasing the impact during negative ones.

In the distraction strategy, as well as under cognitive reappraisal strategy, the participant observed the picture for 1,000 ms, followed by the presentation of the picture and the watermark with a mathematical calculation (all calculations were made up of three operands, including one subtraction and addition). The objective at this point was the participant to solve the calculation while the picture remained on the screen and on the keyboard, it should say whether the calculation answer was correct or incorrect (the letter “c” and “i”, respectively). Both, the picture and the mathematical problem, remained on the screen for 6,000 ms.

The last emotion regulation strategy was the maintain, characterized by the participant passive observation of the picture. After presenting the picture alone in the screen (1,000 ms), participants were presented to a screen with the word “*Look*” (1,000 ms) and then they should only observe the picture without reappraising with a positive or negative perspective (5,000 ms).

As presented on Figure 1, after these 7,000 ms picture presentation period, participants should evaluate the picture situation in two main aspects, its valency and arousal. Using a Brazilian version of the Self-Assessment Manikin (SAM; Bradley and Lang, 1994), participants should first estimate the picture situation in a valence scale from extremely negative (1 point) to extremely positive (9 points), than estimate in a arousal scale from extremely low arousal (1 point) to extremely high arousal (9 points).

Thus, all participants performed 128 trials. Sixteen pictures were used for each of the three emotion regulation strategies (96 pictures for positive and negative pictures). During neutral pictures trials, only distraction and maintain strategies were used (32 pictures for neutral trials). The entire experimental task lasted approximately 35 min.

### Interbeat Interval Measure (IBI)

An ECG signal was recorded via the positioning of two electrodes on the right and left intercostal muscles, and a reference electrode on the anterior/inferior side of the right tibia. Both, data acquisition and data analysis, were performed using BIOPAC^®^ technology with the Acknowledge^®^ software package (Biopac Inc.).

An initial phase of data pre-processing was performed which included: (a) 1,000 μS/V gain; (b) a 0.05 Hz filter (high-pass); and (c) a 35 Hz filter (low-pass). After the detection of markers related to the presentation of emotional images from each regulation category, a time recording was obtained for the cardiac Interbeat Interval (IBI) over the course of the whole experiment, following Gunther Moor et al. (2010) and Marques et al. (2018). IBI values for nine separate points were obtained through this procedure (with 1,000 ms intervals between each IBI value). The IBI values were categorized as: one point occurring prior to target image presentation (IBI-1); one point occurring at the exact moment of the start of target image presentation (IBI-0); and seven points occurring after the start of the target image presentation (IBI-1–IBI-7). To perform data correction relative to the baseline value, the mean value of IBI-1 (baseline) was subtracted from all the IBI values between IBI-0 and IBI-7. As such, the IBI values reported in the results represent the delta values obtained in this procedure.

### Data Analysis

Statistical analyses were conducted using Statistica^®^ software (Stat-Soft Inc., version 8.0). Initially, in order to evaluate the homogeneity of the groups we performed a one-way analysis of variance (ANOVA) on each of the pre-test scales. Next, the scores on positive and negative affect were separately analyzed using repeated-measures ANOVA, using the experimental group (Meditation) and Time (before and after the experimental paradigm) as factors. Regarding the Emotional Experience we performed separate repeated-measures ANOVA for each emotional valence (negative, positive and neutral pictures) and emotional dimension (valence and arousal) with the experimental group and Strategy (Reappraisal, Distraction, and Maintain) as the factors. Finally, a repeated-measures ANOVA was performed for all IBI values using the experimental group, Strategy, and IBI event (IBI-1 to IBI-7) as the factors. Where a significant difference was found between the factors, Student *t*-test was used.

## Results

### Pre-test Scales

A one-way ANOVA revealed no statistical differences for any of the scales (see Table 1).

**Table 1:** Statistical analysis of each scale/questionnaire for each experimental group.

	G1	G2	G3	F	*p*
Age	22.30 (0.65)	21.80 (0.65)	21.70 (0.65)	0.24	0.79
BAI	10.63 (1.29)	8.80 (1.29)	8.53 (1.29)	0.79	0.46
BDI	7.20 (1.14)	10.47 (1.14)	8.83 (1.14)	2.04	0.14
ERQ-CR	29.90 (1.24)	28.77 (1.24)	27.70 (1.24)	0.78	0.46
ERQ-S	15.37 (0.99)	15.30 (0.99)	14.73 (0.99)	0.12	0.88
PMS-AC	1.37 (0.12)	1.39 (0.12)	1.47 (0.12)	0.19	0.83
PMS-AW	2.49 (0.12)	2.67 (0.12)	2.63 (0.12)	0.71	0.49
PANAS-NEG	16.23 (1.00)	16.57 (1.00)	17.20 (1.00)	0.24	0.79
PANAS-POS	32.60 (1.18)	31.53 (1.18)	32.63 (1.18)	0.28	0.76

*The values for each group represent mean and standard error, as well as the *F*- and *p*-values*.

### Effect of Meditations and Emotion Regulation Task on Affect

The levels of positive and negative affect were analyzed separately. For levels of positive affect, a repeated-measure ANOVA revealed a significant main effect of Time [*F*_2.174_ = 43.39; *p* < 0.01; η_p_ = 0.33) but not of **Group** (*F*_2.87_ = 0.43; *p* = 0.65; η_p_ = 0.01), or the interaction between **Group**^∗^Time (*F*_4.174_ = 1.48; *p* = 0.21; η_p_ = 0.03). With respect to the significant main effect observed for Time, lower levels of positive affect following the experimental task (26.94 ± 0.78) were observed as compared to both the baseline (32.26 ± 0.68; *p* < 0.01) and the experimental task (31.34 ± 0.79; *p* < 0.01). No difference was found on the comparison between positive affect levels prior to the intervention and after the intervention (*p* = 0.38).

With respect to the analysis of levels of negative affect, a repeated-measures ANOVA revealed a significant main effect for Time (*F*_2.174_ = 27.17; *p* < 0.01; η_p_ = 0.24) but not for **Group** (*F*_2.87_ = 2.99; *p* = 0.06; η_p_ = 0.06), or the interaction between **Group**^∗^Time (*F*_4.174_ = 0.66; *p* = 0.62; η_p_ = 0.01). With respect to the significant main effect observed for Time, it was not observed significant differences of negative affect levels comparing the moment prior to the intervention (16.67 ± 0.58) and after the experimental task (17.61 ± 0.61; *p* = 0.26). However, it was observed a significant effect after the intervention (12.93 ± 0.35; *p* < 0.01) when compared to the other two moments; i.e., after the 20 min of intervention (independently of the group) a significant decrease of negative affect was observed.

### Effect of Emotion Regulation and Meditation on Emotional Experience

#### Valence

**Figure 2:**
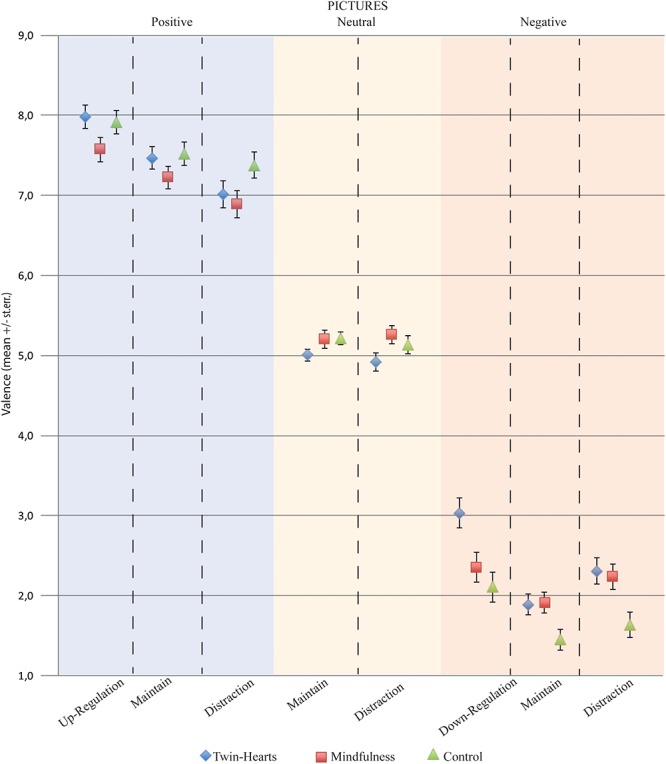
Representation of the mean valence scores by group for negative, positive, and neutral pictures, in respect to the reappraisal, distraction, and maintain strategies.

First, in respect to negative pictures, a repeated-measures ANOVA was conducted on the scores obtained from the emotional valence estimation, revealing a significant main effect of Strategy (*F*_2.174_ = 29.07; *p* < 0.01; η_p_ = 0.25), Group (*F*_2.87_ = 7.17; *p* < 0.01; η_p_ = 0.14), and the interaction Strategy^∗^Group (*F*_4.174_ = 2.68; *p* = 0.03; η_p_ = 0.06). Student *t*-test demonstrates that performing reappraisal (down-regulation), G1 (3.03 ± 0.19), presented significant (*p* = 0.02) higher scores (more positive) as compared to G3 (2.11 ± 0.19; *p* < 0.01), and G2 (2.36 ± 0.19; *p* = 0.02). No significant effect was observed comparing G2 and G3 (*p* = 0.22). In respect to distraction strategy, G1 (2.31 ± 1.07) and G2 (2.23 ± 0.92) presented higher scores (*p* < 0.01) than G3 (1.64 ± 0.54). The same effect was observed for maintain strategy; G1 (1.89 ± 0.95), and G2 (1.92 ± 0.66) presented higher scores as compared to G3 (1.46 ± 0.47; *p* < 0.05). Relative to the use of distraction and maintain strategies, G1 and G2 presented the same scores (*p* > 0.76). Thus, these findings demonstrate that both active interventions resulted in less negative valence when evaluating negative images, and most important only G1 demonstrated significant differences on cognitive reappraisal (Figure 2).

Relative to positive pictures, a repeated-measures ANOVA was conducted on the scores obtained from the emotional valence estimation revealing a significant main effect of Strategy (*F*_2.174_ = 87.11; *p* < 0.01; η_p_ = 0.50), and the interaction Strategy^∗^Group (*F*_2.87_ = 2.87; *p* = 0.02; η_p_ = 0.06), but not for Group (*F*_2.87_ = 1.81; *p* = 0.17; η_p_ = 0.04). Independent *t*-tests demonstrated that G1 (7.98 ± 0.72) and G2 (7.57 ± 0.85) were significant different between them (*p* < 0.05) in respect to the use of reappraisal, but both groups did not differ from G3 (*p* > 0.12). In contrast, under distraction strategy, G2 (6.89 ± 0.93) significantly differ (*p* = 0.04) from G3 (7.38 ± 0.92), but both groups did not differ (*p* = 0.59) from G1 (7.02 ± 0.91), being G2 more effective to perform distraction compared to G3.

Finally, repeated-measures ANOVA did not find any significant effect for neutral pictures, considering the factor Strategy (*F*_1.87_ = 0.43; *p* = 0.52; η_p_ < 0.01), Group (*F*_2.87_ = 2.98; *p* = 0.06; η_p_ = 0.06) and the interaction Strategy^∗^Group (*F*_2.87_ = 0.52; *p* = 0.59; η_p_ = 0.01). These results demonstrate that neutral pictures were similarly evaluated irrespective of meditation intervention and emotion regulation strategy.

#### Arousal

**Figure 3:**
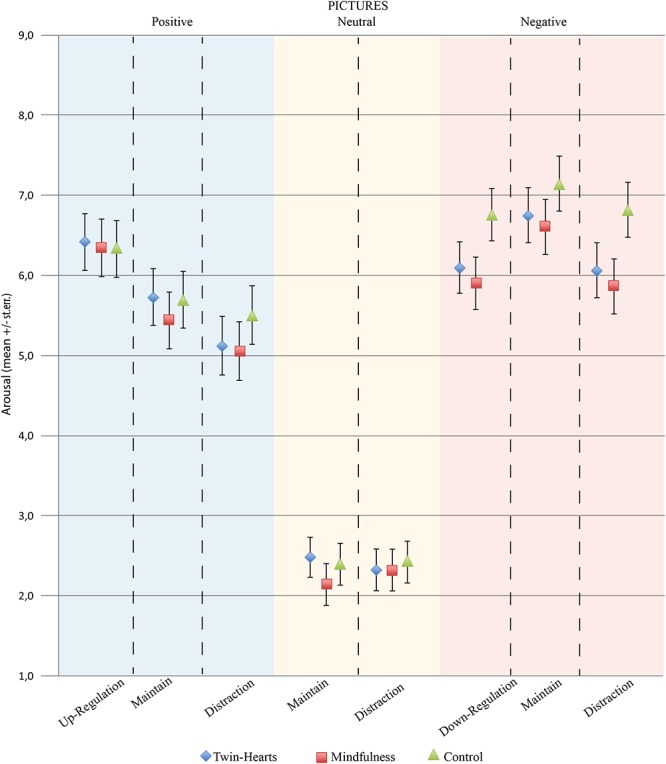
Representation of the mean arousal scores by groups for negative, positive, and neutral pictures, in respect to the reappraisal, distraction, and maintain strategies.

Relative to Arousal estimation, and specifically to negative pictures, a repeated-measures ANOVA was conducted revealing a significant main effect of Strategy (*F*_2.87_ = 30.93; *p* < 0.01; η_p_ = 0.26), but not for Group (*F*_2.87_ = 2.58; *p* = 0.22; η_p_ = 0.04), and the interaction Strategy^∗^Group (*F*_2.87_ = 22.26; *p* = 0.29; η_p_ = 0.03). Thus, considering the main effect of Strategy, independent Student *t*-tests demonstrated that reappraisal (6.25 ± 1.79) and distraction (6.25 ± 1.91) presented lower scores (*p* < 0.04) than maintain strategy (6.83 ± 1.87), but without significant differences between them (*p* < 0.99). In this fashion, this finding demonstrates that both active interventions resulted in less arousal when evaluating negative images (Figure 3).

In respect to positive pictures, repeated-measures ANOVA was conducted revealing a significant main effect of Strategy (*F*_2.274_ = 95.57; *p* < 0.01; η_p_ = 0.52), but not for Group (*F*_2.87_ = 0.22; *p* = 0.89; η_p_ = 0.00), and the interaction Strategy^∗^Group (*F*_4.274_ = 2.04; *p* = 0.09; η_p_ = 0.04). In this sense, after performing independent Student *t*-tests it was possible to assume that all emotion regulation strategies were different from each other (*p* < 0.01), except comparing maintain and distraction scores (*p* = 0.18). Thus, reappraisal strategy (6.38 ± 1.93) presented lower levels of arousal estimation compared to distraction (5.23 ± 2.00) and maintain (5.63 ± 1.92). These results indicate greater effectiveness of reappraisal compared to distraction.

Finally, in the same way as for valence scores, neutral pictures did not present any significant effect considering the factor Strategy (*F*_1.87_ = 0.04; *p* = 0.83; η_p_ < 0.01), and Group (*F*_2.87_ = 0.20; *p* = 0.82; η_p_ < 0.01), as well as for the interaction Strategy^∗^Group (*F*_2.87_ = 1.81; *p* = 0.17; η_p_ = 0.04). Again, these results demonstrate that neutral pictures were similarly evaluated irrespective of meditation intervention and emotion regulation strategy.

### Effect of Emotion Regulations and Meditation on Interbeat Interval (IBI)

**Figure 4:**
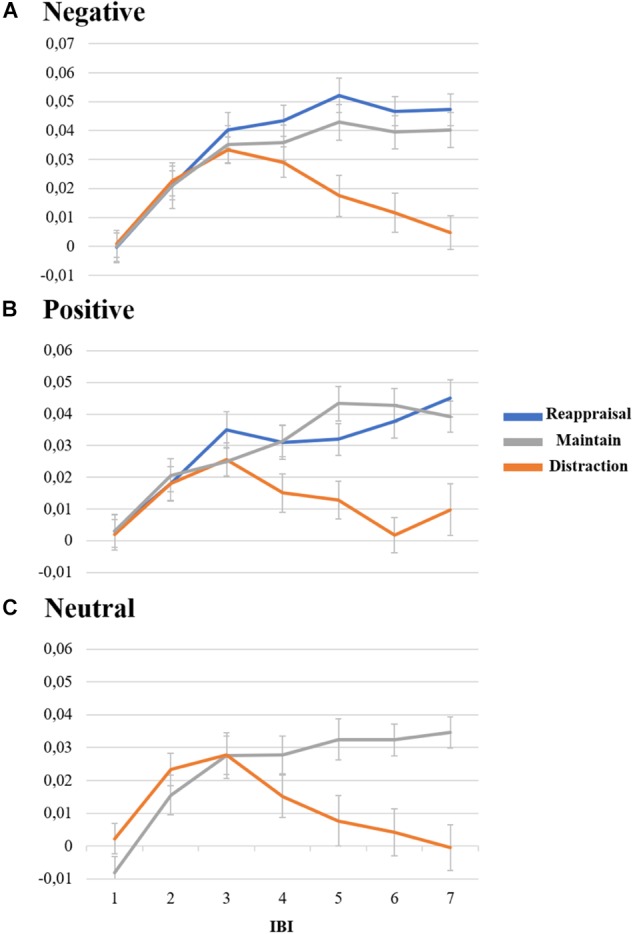
Representation of IBI variation during the task of emotion regulation. Lines are divided by strategy, where values represent IBI delta value, and spreads represent standard error. **(A)** Value of IBI for negative pictures. **(B)** Value of IBI for positive pictures. **(C)** Value of IBI for neutral pictures.

As performed for the emotional experience estimations, three repeated measures ANOVA were performed for each group of pictures (negative, positive, and neutral). First, considering negative pictures, repeated measures ANOVA revealed significant main effect for Strategy (*F*_2.174_ = 5.64; *p* < 0.01; η_p_ = 0.06), Time (*F*_6.516_ = 27.69; *p* < 0.01; η_p_ = 0.24), and the interaction Strategy^∗^Time (*F*_12.1032_ = 7.75; *p* < 0.01; η_p_ = 0.08), but not for Group (*F*_2.86_ = 1.54; *p* = 0.22; η_p_ = 0.03), the interaction Strategy^∗^Group (*F*_4.172_ = 1.99; *p* = 0.10; η_p_ = 0.04), Time^∗^Group (*F*_12.516_ = 0.81; *p* = 0.64; η_p_ = 0.02), and the interaction Strategy^∗^Time^∗^Group (*F*_24.1032_ = 0.83; *p* = 0.70; η_p_ = 0.02). Considering the interaction between Strategy and Time, Student *t*-test revealed that only the IBI values related to the distraction strategy significantly varies (*p* < 0.01) from reappraisal and maintain on IBI-5 to IBI-7 (see Figure 4A).

Considering positive pictures, and similar to negative pictures results, repeated measures ANOVA revealed significant main effect for Strategy (*F*_2.172_ = 7.20; *p* < 0.01; η_p_ = 0.08), Time (*F*_6.516_ = 15.06; *p* < 0.01; η_p_ = 0.15), and the interaction Strategy^∗^Time (*F*_12.1032_ = 6.94; *p* < 0.01; η_p_ = 0.07), but not for Group (*F*_2.86_ = 0.10; *p* = 0.90; η_p_ = 0.00), the interaction Strategy^∗^Group (*F*_4.172_ = 0.07; *p* = 0.99; η_p_ ≤ 0.01), Time^∗^Group (*F*_12.516_ = 0.49; *p* = 0.92; η_p_ = 0.01), and the interaction Strategy^∗^Time^∗^Group (*F*_24.1032_ = 1.10; *p* = 0.33; η_p_ = 0.03). Again, Bonferroni *post hoc* revealed significant differences from reappraisal compared to distraction (*p* < 0.01) but not to maintain strategy (*p* = 1.00), However, contrary to negative pictures results, distraction significantly differed from maintain strategy (*p* < 0.01). Considering the interaction between Strategy and Time, Student *t*-test revealed that only the IBI values related to the distraction strategy significantly varies from reappraisal and maintain on IBI-5 to IBI-7 (*p* < 0.01; see Figure 4B).

Lastly, it was performed a repeated measures ANOVA for neutral pictures, which only revealed significant differences for Time (*F*_6.516_ = 10.01; *p* < 0.01; η_p_ = 0.10), and the interaction Strategy^∗^Time (*F*_6.516_ = 9.99; *p* < 0.01; η_p_ = 0.10), but not for Strategy (*F*_1.86_ = 3.91; *p* = 0.05; η_p_ = 0.04), Group (*F*_2.87_ = 0.02; *p* = 0.98; η_p_ < 0.01), the interaction Strategy^∗^Group (*F*_2.86_ = 0.45; *p* = 0.64; η_p_ = 0.01), Time^∗^Group (*F*_12.516_ = 1.29; *p* = 0.22; η_p_ = 0.03), and the interaction Strategy^∗^Time^∗^Group (*F*_12.516_ = 0.64; *p* = 0.80; η_p_ = 0.01). Additionally, Student *t*-test was performed demonstrating significant differences between strategies for IBI-5, IBI-6, and IBI-7 (Figure 4C), which revealed that under distraction strategy participants presented significant lower IBI values (higher cardiac recruitment) than under maintain strategy (*p* = 0.01). Figure 4 summarize these IBI results for all pictures valences.

## Discussion

The present study aimed to investigate the immediate effect of two different types of meditation (MF and THM) on cognitive reappraisal and distraction. We structured our discussion in the following themes: *effects of meditation*; *effects of emotion regulation strategies*; and *task Effectiveness*.

### Effects of Meditation

Mindfulness meditation (MF), a practice that aims to refine the process of turning attention to the present moment (Kabat-Zinn, 2015), has been defined in current research as a powerful therapeutic tool to promote health and well-being benefits in the people’s life (Lin et al., 2016). One of these benefits is often related to refinement of the control and regulation of emotions (Lutz et al., 2008). Thus, this study had the expectation of finding greater emotional control by the participants who practiced MF, since this is the only practice of this study which has a theoretical basis in this sense. From the analyzes of the emotional valence judgments of the images, it was possible to find differences between the types of intervention in the emotion regulation. However, differently than it was expected, the participants who practiced THM were more effective in suppressing the negative emotion and amplifying the positive emotion than those who practiced MF and the control group, which may be related to the greater effect of this type of meditation on emotional control.

This result can be justified from understanding the content of THM. During all meditation, the participant was invited to think and wish something positive for the **Earth and for all humanity**. Although this meditation is little known and does not have scientific literature to operationalize it, its content resembles other more popular meditations belonging to group of KBM. According to He et al. (2015), LKM, a contemplative meditation aimed to cultivating positive thoughts about one another and oneself, typically increases positive emotions and decreases negative ones. These characteristics are similar to the THM practiced by G1 in the present study, which may justify the better performance of this group in increasing or decreasing emotions. Therefore, one question must be asked: why MF has not had that same effect on emotion regulation?

Twin Hearts Meditation leads the practitioner’s attention precisely to the expansion of positive emotions, which closely resembles the up-and down regulation strategy in which this group was most effective (reappraisal). MF practice, in turn, encourages the practitioner to direct attention to the thoughts that came to consciousness and then let them go, without being distracted by their content. At first, we can identify the similarity between the two practices: both of them regulate attention for a given stimulus. This attentional training, which is inherent to the meditation practices mentioned here, was possible to be noticed in this study through the better performance of these two groups as a function of the control group when compared to the distraction strategy. Indeed, the attentional training provided by meditations, regardless of their type, influenced emotion regulation from a strategy of attention directed. On the other hand, in relation to the cognitive reappraisal strategy, THM was more effective than MF, as previously exposed. Our findings introduce an important variable when choosing a meditation practices: the content of meditation matters on the emotion regulation, at least when we seek to investigate the immediate effect of meditation. Future studies might address the effects of short versus long term practices as well as the last-long effects.

One limitation of our study is related to some differences between the meditation’s audios. We administered the instructional audios in their original format strictly following the procedures used by the instructors, i.e., we kept everything as it was originally recorded including the length of each video and background sounds. Thus, the THM intervention had 27 min long with background calm music while MF had 20 min and no music. These two differences might had impacted some of our findings. New studies are needed to test for the effects of length and background music.

In addition to the results found in relation to reappraisal and distraction, another finding draws our attention. In the maintain strategy, that is, when the participant responded spontaneously during passive observation of the stimuli, both groups of meditation were reported less negative emotional arousal as compared to the control group. At the same time, no effect was observed for valence evaluation during passive observation. Thus, going through a meditation practice (independently of its type) reduced the arousal evoked by the stimuli while kept the same judgment of valence.

Regarding the variation of the negative affect of the participants (measured by PANAS), it was observed after the 20 min of MF, THM or control group the negative affect was decreased. Thus, our results do not point to significant effects of any type of meditation on affect modulation when it is practice for the first time in one session only. It is important to observe that that long-term experience of meditation can lead to more satisfactory results on the modulation of affect (Garland et al., 2009; Hölzel et al., 2011).

### Effects of Emotion Regulation Strategies

The intensity evaluations of the positive and negative pictures, performed after the use of each of the strategies clearly demonstrate the specificities of each one of them in the modulation of the emotional response components. In respect of negative images, down-regulation and distraction were equally effective in suppressing emotional intensity than maintain. These results indicate that both strategies were adequately performed by the participants, in addition to comply their objective: to change the current emotional arousal (Gross and Thompson, 2006). However, when dealing with the arousal judgment for positive pictures, it is possible to identify the differences between the two strategies, a topic that has already been discussed here. If to suppress an emotion, distraction is effective, however, if the main goal is to amplify this emotion, this strategy is not the best choice. This is because their goal is to shift the attentional focus of the emotional stimulus to an alternative stimulus (Sheppes et al., 2011). In this sense, it is not possible to amplify an emotion without coming into contact with it. For this purpose, the cognitive reappraisal strategy is more effective, as it was possible to observe in the results of the present study. These results corroborate with the literature (Sheppes and Gross, 2011) and point out the importance that the different strategies of emotion regulation continue to be studied.

Regarding the *psychophysiological effects*, although no significant group effect was found in the interbeat intervals (IBI), the results of the electrocardiographic record (ECG) contribute to this discussion about the particularities of each of the emotion regulation strategies. Regardless of the group or the valence of the image, the *distraction* strategy recruited greater cardiac acceleration in the intervals 5, 6, and 7, corresponding to the last 2,000 ms of image presentation (time of the use of the strategy). At the same time, the *cognitive reappraisal* strategy presented a decrease in cardiac acceleration, as well as the strategy of *passive observation*. These results point once again to the differences between strategies. Thus, it is evident that the strategy of distraction, which in the case of this study was based on presenting a arithmetic calculation before the emotional image, differentiated from others strategies, recruiting more cardiac activity. This data corresponds to recent results found by Monaco et al. (2017), in which arithmetic activities recruited accelerated cardiac activity. In this sense, the result found in the present study can be justified by the content of the activity to which the participant directed his attention. Even though it was made clear for participants that their hits and misses would not be recorded, the pressure and stress that a arithmetic calculation typically generates could be expressed by the ECG results.

### Task Effectiveness

Finally, regarding the levels of positive affect throughout the experiment, regardless of the group, the positive affect of the participants decreased after exposure to the task of emotion regulation. This data indicates that the task used in the experiment complied with the objective of eliciting emotions through pictures of negative affect. Thus, through the PANAS values, the experimental design developed by Kanske et al. (2010) and adapted for this study, as well as all the emotional stimuli used, once again proved to be efficient to induce negative emotions.

## Conclusion

This study, which aimed to investigate the effect of meditative practices on the cognitive ability of emotion regulation, presented corroborative results with the literature. Regarding the meditations, both types discussed here presented significant effects in the use of the distraction strategy. Thus, it is possible to say that a single meditative practice, be it mindfulness-based or positive emotional-based, is capable of enhancing attentional skills. On the other hand, when it comes to cognitive reappraisal, a single THM experience has proved sufficient to enhance the ability to amplify or suppress emotional arousal, a result not found in relation to MF. In this sense, it seems that the content of the thoughts during the meditation matters, i.e., a positive emotional-based meditation can be used as an efficient tool to produce immediate effect in the emotional modulation.

Finally, regarding the strategies of emotional regulation adopted in this study, it became clear that this topic should continue to gain prominence in the next researches, since both strategies chosen proved to be efficient in emotional control. It is also important for future studies to continue to adopt tasks in which different types of strategies are compared, since, according to the previous literature and with the results of this study, each one has specificities and, therefore, behave differently in modulating the components of emotional response.

## Data Availability

The datasets generated for this study are available on request to the corresponding author.

## Ethics Statement

This study was carried out in accordance with the recommendations of CONEP, CEP-Mackenzie with written informed consent from all subjects. All subjects gave written informed consent in accordance with the Declaration of Helsinki. The protocol was approved by the CEP-Mackenzie (SISNEP, Brazil; CAAE: 79465017.7.0000.0084).

## Author Contributions

CV and PB developed the study concept and design. Data collection was performed by CV. CV, LM, and PB performed the data analysis. All authors contributed to the data interpretation, manuscript writing, and approved the final version of the manuscript for submission.

## Conflict of Interest Statement

The authors declare that the research was conducted in the absence of any commercial or financial relationships that could be construed as a potential conflict of interest.
