# Supplementary material for: A Positive Emotional-Based Meditation but Not Mindfulness-Based Meditation Improves Emotion Regulation
Source: Front Psychol. 2019 Mar 28;10:647. doi: 10.3389/fpsyg.2019.00647 (PMC6448484; doi:10.3389/fpsyg.2019.00647)
Supplement: Supplementary file 1 [file Table_1.DOCX]

**SUPPLEMENTARY MATERIAL**

|  | ***Negative images*** |  |
| --- | --- | --- |
| **IAPS number** | **Valence mean (SD)** | **Arousal mean (SD)** |
| 2703 | 1.91(1.26) | 5.78(2.25) |
| 2800 | 1.78(1.14) | 5.49(2.11) |
| 3010 | 1.79(1.28) | 7.26(1.86) |
| 3015 | 1.52(0.95) | 5.90(2.82) |
| 3060 | 1.79(1.56) | 7.12(2.09) |
| 3080 | 1.48(0.95) | 7.22(1.97) |
| 3350 | 1.88(1.67) | 5.72(2.23) |
| 6212 | 2.19(1.49) | 6.01(2.44) |
| 9252 | 1.98(1.59) | 6.64(2.33) |
| 9253 | 2.00(1.19) | 5.53(2.40) |
| 9254 | 2.03(1.35) | 6.04(2.35) |
| 9410 | 1.51(1.15) | 7.07(2.06) |
| 9635,1 | 1.90(1.31) | 6.54(2.27) |
| 9910 | 2.06(1.26) | 6.20(2.16) |
| 9921 | 2.04(1.47) | 6.52(1.94) |
| 3230 | 2.02(1.30) | 5.41(2.21) |
|  | ***Positive images*** |  |
| **IAPS number** | **Valence mean (SD)** | **Arousal mean (SD)** |
| 2224 | 7.24(1.58) | 4.85(2.11) |
| 2340 | 8.03(1.26) | 4.90(2.20) |
| 4608 | 7.07(1.66) | 6.47(1.96) |
| 4626 | 7.60(1.66) | 5.78(2.42) |
| 4660 | 7.40(1.36) | 6.58(1.88) |
| 4687 | 6.87(1.51) | 6.51(2.10) |
| 4689 | 6.90(1.55) | 6.21(1.74) |
| 4695 | 6.84(1.53) | 6.61(1.88) |
| 5621 | 7.57(1.42) | 6.99(1.95) |
| 5629 | 7.03(1.55) | 6.55(2.11) |
| 8030 | 7.33(1.76) | 7.35(2.02) |
| 8190 | 8.10(1.39) | 6.28(2.57) |
| 8200 | 7.54(1.37) | 6.35(1.98) |
| 8370 | 7.77(1.29) | 6.73(2.24) |
| 8380 | 7.56(1.55) | 5.74(2.32) |
| 8490 | 7.20(2.35) | 6.68(1.97) |
|  | ***Neutral images*** |  |
| **IAPS number** | **Valence mean (SD)** | **Arousal mean (SD)** |
| 2102 | 5.16(0.96) | 3.03(1.87) |
| 2190 | 4.83(1.28) | 2.41(1.80) |
| 2215 | 4.63(1.24) | 3.38(2.00) |
| 2383 | 4.72(1.36) | 3.41(1.83) |
| 2393 | 4.87(1.06) | 2.93(1.88) |
| 2396 | 4.91(1.05) | 3.34(1.83) |
| 2397 | 4.98(1.11) | 2.77(1.74) |
| 2440 | 4.49(1.03) | 2.63(1.70) |
| 2480 | 4.77(1.64) | 2.66(1.78) |
| 2495 | 5.22(1.10) | 3.19(1.76) |
| 2570 | 4.78(1.24) | 2.76(1.92) |
| 2745,1 | 5.31(1.08) | 3.26(1.96) |
| 2840 | 4.91(1.52) | 2.43(1.82) |
| 2870 | 5.31(1.41) | 3.01(1.72) |
| 7493 | 5.35(1.34) | 3.39(2.08) |
| 9210 | 4.53(1.82) | 3.08(2.13) |
